# Inferring Selection Intensity and Allele Age from Multilocus Haplotype Structure

**DOI:** 10.1534/g3.113.006197

**Published:** 2013-08-01

**Authors:** Hua Chen, Montgomery Slatkin

**Affiliations:** Department of Integrative Biology, University of California, Berkeley, California 94720

**Keywords:** selection coefficient, allele age, haplotype structure, structured coalescent, importance sampling, time-varying population size

## Abstract

It is a challenging task to infer selection intensity and allele age from population genetic data. Here we present a method that can efficiently estimate selection intensity and allele age from the multilocus haplotype structure in the vicinity of a segregating mutant under positive selection. We use a structured-coalescent approach to model the effect of directional selection on the gene genealogies of neutral markers linked to the selected mutant. The frequency trajectory of the selected allele follows the Wright-Fisher model. Given the position of the selected mutant, we propose a simplified multilocus haplotype model that can efficiently model the dynamics of the ancestral haplotypes under the joint influence of selection and recombination. This model approximates the ancestral genealogies of the sample, which reduces the number of states from an exponential function of the number of single-nucleotide polymorphism loci to a quadratic function. That allows parameter inference from data covering DNA regions as large as several hundred kilo-bases. Importance sampling algorithms are adopted to evaluate the probability of a sample by exploring the space of both allele frequency trajectories of the selected mutation and gene genealogies of the linked sites. We demonstrate by simulation that the method can accurately estimate selection intensity for moderate and strong positive selection. We apply the method to a data set of the *G6PD* gene in an African population and obtain an estimate of 0.0456 (95% confidence interval 0.0144−0.0769) for the selection intensity. The proposed method is novel in jointly modeling the multilocus haplotype pattern caused by recombination and mutation, allowing the analysis of haplotype data in recombining regions. Moreover, the method is applicable to data from populations under exponential growth and a variety of other demographic histories.

## Introduction

There is an increased interest in elucidating the role of natural selection in the evolution of human and other species using population genetic data. Evidence shows that selection has been actively shaping the genetic diversity of human populations during the process of adaptation to new environments and infectious diseases (Sabeti *et al.* 2002; Bersaglieri *et al.* 2004; Tishkoff *et al.* 2007; Simonson *et al.* 2010; Yi *et al.* 2010; Peng *et al.* 2011; Xu *et al.* 2011; Kamberov *et al.* 2013). Selection in human populations can leave “footprints” in patterns of single-nucleotide polymorphisms (SNPs) in the vicinity of the selected mutant. Numerous methods have been developed to detect natural selection based on such polymorphism patterns (Tajima 1989; Fu and Li 1993; Fay and Wu 2000; Kim and Stephan 2002; Sabeti *et al.* 2002; Nielsen *et al.* 2005; Voight *et al.* 2006; Sabeti *et al.* 2007; Tang *et al.* 2007; Chen *et al.* 2010). However, only a few methods are available for inferring quantities of the selective process, such as selection intensity and allele age. Among the existing methods, some consider single markers linked to the selected locus (*e.g.*, Slatkin 2001; Kim and Stephan 2002), whereas more sophisticated methods gain information by exploiting the haplotype structure of multiple marker loci. For example, Coop and Griffiths (2004) inferred selection intensity and allele age by analyzing mutations among different haplotypes along their genealogical history. Coop and Griffiths (2004) used an importance sampling algorithm to explore possible gene genealogies. Recombination is not allowed by their method, and thus it works only for nonrecombining regions. Rannala and Reeve (2004) extended their former likelihood approach for disease mapping (Rannala and Reeve 2001) to estimate allele age of a mutant under neutrality using multiple linked markers, and employed Markov Chain Monte Carlo to generate the posterior distribution of allele age of the mutant. Slatkin (2008) presented a Bayesian method for jointly inferring selection intensity and allele age of the mutant using linkage disequilibria of multiple marker loci and generated the probability of data with an importance sampling algorithm.

The aforementioned multilocus methods all require modeling the effect of selection on the genealogical structure of neutral markers under a coalescent framework, which can be done in two ways. The first approach is to use the Krone-Neuhauser ancestral selection graph (ASG; Krone and Neuhauser 1997). In the ASG, the genealogy of the selected allele is embedded in a branching-coalescing graph so that both selection and mutation can be incorporated in the graph. The ASG approach is useful for simulating genealogies under weak selection. For moderate or strong selection, the ASG becomes so large that the computation becomes intractable. The ASG method’s performance was dramatically improved by truncating the ASG (Slade 2000) to avoid generating very large ASGs. This approach, however, has not been extended to the analysis of multiple linked neutral mutations. The second approach is the structured coalescent (Kaplan *et al.* 1988; Hudson and Kaplan 1988), which generates historical frequency trajectories of the selected allele and then treats chromosomes carrying the mutant allele and nonmutant allele as subpopulations between which there exists “gene flow” caused by recombination. For alleles under balancing selection, the allele frequencies were assumed to be constant. For positively selected alleles, the allele frequency trajectories can either be generated by stochastic simulation or be approximated using deterministic equations.

The aforementioned multilocus methods all adopted the structured-coalescent model of selection to estimate the selection parameters (Coop and Griffiths 2004; Slatkin 2008), which is also the model used in our proposed method. But the approach to sampling the allele frequency trajectory from its probability distribution in our method differs from these others. Coop and Griffiths (2004) generated random trajectories of selected mutations under the Moran model, which has the property of time reversibility under mutation and additive selection (Watterson 1975) but works only for populations of constant size. Rannala and Reeve (2004) made an assumption that the historical allele frequencies of neutral markers are constant during the whole process, which may not hold for real populations, especially for markers under the hitch-hiking effect. Slatkin (2008) used a linear birth-and-death process to approximate the genealogical trees of haplotypes carrying selected mutants, which is an adequate approximation for mutants in low frequency, but may not be suitable for common mutants. We use the Wright-Fisher model instead of the Moran model to generate allele frequency trajectories, and apply the importance sampling scheme in Slatkin (2001) to weight the trajectories when estimating selection parameters. This allows us to model the selective sweeps in a population with time-varying size, and allows us to analyze both high- and low-frequency alleles under selection.

One advantage of the proposed method over the existing methods is that we model the dynamics of ancestral haplotypes under the joint effects of selection, mutation and recombination during a selective sweep process. In contrast, Coop and Griffiths (2004) assumed no recombination in their model, whereas both Slatkin (2008) and Rannala and Reeve (2004) simplified the transitions among different multi-loci haplotypes induced by recombination. In particular, some of the recombination events between different types of haplotypes within the selected haplotype group were ignored. This restriction can cause significant bias when the selected allele is in medium or high frequency in the population. Our method explicitly describes the frequencies of different selected haplotypes over time during the selective process. Therefore, our proposed method applies to both high- and low-frequency mutants. Our method also requires some approximations to improve computational efficiency.

As we will demonstrate in the section *A simplified multi-locus model for haplotype structure*, the model efficiently reduces the state space of ancestral haplotypes from an exponential function of the number of SNP loci to a quadratic function, and thus allows the inference of allele age and selection intensity from multi-SNP haplotypes spanning several hundred kilo-bases or even mega-bases affected by strong selections. We then modify the importance sampling method of Griffiths and Tavaré (1994b) to obtain the probability of a sample configuration and to estimate the selection parameters by averaging over genealogies of the linked sites. Note that an alternative choice is to adopt the existing importance sampling algorithms developed for multilocus ancestral recombination graph (ARG) under neutrality (Griffiths and Marjoram 1996; Fearnhead and Donnelly 2001) and incorporate the ARG into the structured-coalescent model. However, because of the large state space of genealogies that has to be explored by the importance sampling algorithms for a multilocus ARG, this approach is intractable on a genomic scale of hundreds of kilobases.

## Overview of the Method

**Figure 1:**
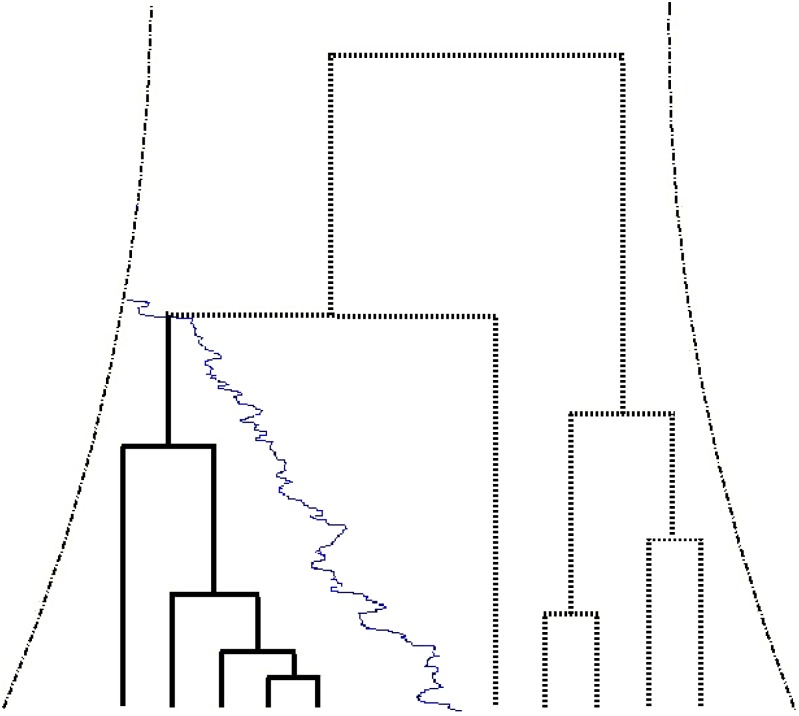
An illustration of the structured-coalescent approach for modeling positive selection. The historical population sizes are indicated by the distance between the two dashed lines; and the allele frequency trajectory of the selected allele is indicated with a thin solid curve. The coalescent history of the selected locus with five derived lineages (solid bold lines) and five ancestral lineages (dotted bold lines) is superimposed on the trajectory and population size curves. The present time, *t* = 0, is at the bottom. And the time at which the trajectory of the selected mutant merged to the population-size curve denotes the time when the selected mutant arose in the population, *i.e.*, the allele age *T*. In the model presented in the main text, only the sub-genealogies in the selected allele groups (bold solid lines) are considered.

Suppose the data consist of *n*_sample_ haplotypes with known phase collected from the current population. If genotype data are collected, the phase can be estimated by available algorithms (Scheet and Stephens 2006). The haplotypes are divided into two groups: the selected haplotypes, which are the chromosomes carrying the selected allele, and the background haplotypes, which do not carry the selected allele. In this method, we view the coalescent process in the *n* selected haplotypes as in a structured subpopulation (see Figure 1). The *n* selected haplotypes are represented by a *n* by *m* matrix with “1” or “0” for each entry, D*_i_*_,_*_j_*, corresponding to the allele type of the *i*th haplotype at the *j*th SNP position. The position of the selected mutant is assumed to be known, as is the genetic distance between the *j*_1_th and *j*_2_th SNP, {*r_j_*_1_*_j_*_2_, 1 ≤ *j*_1_, *j*_2_ ≤ *m*}.

We model the effect of selection with the structured-coalescent scheme (see Figure 1). The likelihood function of the observed selected haplotype data, D, can be computed from

ℒ(s)=ℙ(D|s,Γ)=∬ℙ(D|G)ℙ(G|ℋ)ℙ(ℋ|s,Γ)dGdℋ, (1)

where G denotes the genealogy and ℋ the frequency trajectory of the selected mutant. Neither ℋ nor G is observed directly. When constructing the likelihood function, they are often integrated out (Felsenstein 1988; Griffiths and Tavaré, 1994b; Kuhner *et al.* 1995).

The frequency trajectory of the selected mutant, ℋ, is a random process that follows the Wright-Fisher model and has a probability distribution, *ℙ(ℋ|s*, *Γ*), that depends on the selection intensity *s* and the nuisance parameter set Γ, which includes all the other parameters related to the population history. Conditional on any given frequency trajectory, the sampling probability of the data is constructed by summing over all possible genealogical events: ℙ(D|ℋ)=∫ℙ(D|G)ℙ(G|ℋ)dG.

For computationally efficient evaluation of the sampling probability, we propose a novel simplified multilocus model for the transition of different types of selected haplotypes (see the section *A simplified multilocus model for haplotype structure*). We compute the extent of the ancestral haplotypes in the vicinity of the selected mutant under the combined effects of recombination and selection. Together with the infinitely-many-sites model for mutations, the simplified multilocus haplotype model is used to approximate the evolutionary dynamics of the data.

**Figure 2:**
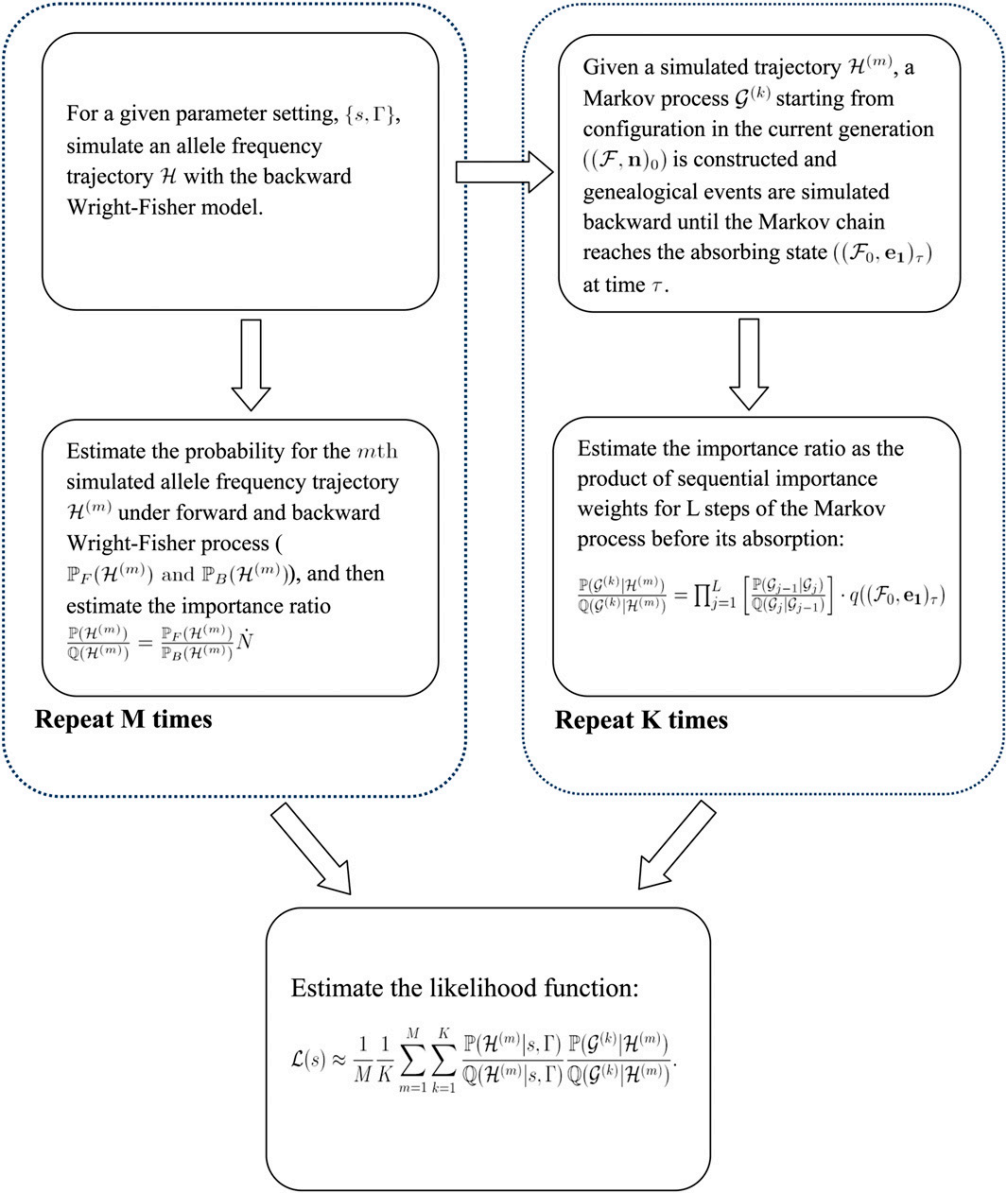
Flowchart of importance sampling procedures of the method.

Because the spaces of both gene genealogies and allele frequency trajectories are too large to explore, ℙ(G|ℋ) and ℙ(ℋ|*s*, Γ) cannot be expressed in closed forms. We use the importance sampling algorithms to sample genealogies and trajectories that are compatible with the data from the proposal distributions. Then the likelihood is estimated as the weighted average probability for the samples. The importance weights are obtained by taking the ratio of the probabilities of true distribution and the proposal distribution. The procedure of evaluating the likelihood is illustrated by the flowchart in Figure 2. Two main steps of the flowchart correspond to sampling ℋ and G using the importance sampling algorithms. The details of the algorithms and calculation of the importance ratios are presented in the section *Importance sampling and proposal distributions*.

## A simplified multilocus model for haplotype structure

We model the transition of the selected haplotypes (haplotypes carrying the selected mutant) under the influence of evolutionary events including recombination and mutation. We start with a sample of selected haplotypes collected from the current generation. When looking backward in time, we can eventually trace these selected haplotypes to one common ancestor (the ancestral haplotype) because all copies of the selected allele are descended from a single mutation. During the selection process, recombination breaks up and mixes the fragments with the background haplotypes. Recombination combined with mutation generates the different selected haplotypes that contain some segments of the ancestral haplotype. The number of distinct selected haplotypes at different times in the history is called the ancestral process. This ancestral process, conditional on the frequency trajectory of the selected mutant, can be viewed as a structured-coalescent process, because the selected haplotypes evolve as a subpopulation of the entire haplotype pool, with the size of the subpopulation determined by the mutant allele frequency, and the transitions among different haplotypes following the simplified multilocus model.

To illustrate the state space of the ancestral process and the joint effect of selection and recombination on the transitions between the ancestral states, we will start with a simple case of only two loci, the selected locus and a partially linked SNP locus. We then extend the two-locus model to a simplified multi-locus haplotype model, after making several approximations for computational efficiency. Then in the section *Sampling probability of a multi-locus haplotype configuration*, the simplified multilocus haplotype model and the infinitely-many-sites model for mutation are used to derive the sampling probability for haplotype configuration of a sample by summing over possible ancestral states of the genealogical history, that is, the $\sum_{G}\mathbb{P}\left(D|G\right)\mathbb{P}\left(G|ℋ\right)$ component of likelihood function conditional on a simulated allele frequency trajectory.

### A two-locus model

The two-locus haplotype model involves only the selected locus and one neutral marker, the positions of which are assumed known. The selected locus has the mutant allele A and the other neutral allele a. The neutral marker locus has two alleles B and b. Let *Q*(*t*) = (*q*_1_, *q*_2_; *q*_3_, *q*_4_) denote the number of haplotypes AB, Ab, aB, and ab in the sample at time *t*. Conditional on the ancestral allele frequency trajectory, {*X_t_*, *t* > 0}, the “ancestral process” *Q*(*t*), which is defined as the numbers of each haplotype, can be approximated by the inhomogeneous Markov process (Hudson and Kaplan 1988; Durrett and Schweinsberg 2004). The states that the process *Q*(*t*) can jump from state (*q*_1_, *q*_2_; *q*_3_, *q*_4_) to include (*q*_1_ − 1, *q*_2_; *q*_3_, *q*_4_), (*q*_1_ − 1, *q*_2_ + 1; *q*_3_, *q*_4_), (*q*_1_ − 1, *q*_2_; *q*_3_ + 1, *q*_4_), (*q*_1_ − 1, *q*_2_; *q*_3_, *q*_4_ + 1), (*q*_1_, *q*_2_ − 1; *q*_3_, *q*_4_), (*q*_1_ + 1, *q*_2_ − 1; *q*_3_, *q*_4_), (*q*_1_, *q*_2_ − 1; *q*_3_ + 1, *q*_4_), (*q*_1_, *q*_2_ − 1; *q*_3_, *q*_4_ + 1), (*q*_1_, *q*_2_; *q*_3_ − 1, *q*_4_), (*q*_1_ + 1, *q*_2_; *q*_3_ − 1, *q*_4_), (*q*_1_, *q*_2_ + 1; *q*_3_ − 1, *q*_4_), (*q*_1_, *q*_2_; *q*_3_ − 1, *q*_4_ + 1), (*q*_1_, *q*_2_; *q*_3_, *q*_4_ − 1), (*q*_1_ + 1, *q*_2_; *q*_3_, *q*_4_ − 1), (*q*_1_, *q*_2_ + 1; *q*_3_, *q*_4_ − 1) and (*q*_1_, *q*_2_; *q*_3_ + 1, *q*_4_ − 1). The transition probabilities from (*q*_1_, *q*_2_; *q*_3_, *q*_4_) to the first four states are listed in Table 2, and the other transition probabilities can be constructed similarly. We assume an infinitely-many-sites model for mutations, so there are no new or recurrent mutations between the two alleles of either the selected locus or the neutral marker locus. Let *N_AB_*(*t*), *N_Ab_*(*t*), *N_aB_*(*t*), and *N_ab_*(*t*) be the population counts of the four corresponding haplotypes *AB*, *Ab*, *aB* and *ab* at time *t* respectively. For the transition from (*q*_1_, *q*_2_; *q*_3_, *q*_4_) to (*q*_1_ − 1, *q*_2_; *q*_3_, *q*_4_), no recombination has occurred and two lineages of haplotype AB are chosen to coalesce. The coalescence rate is $q_{1}\left(1-r\right)\frac{q_{1}-1}{N_{AB}\left(t\right)}$; for the transition from (*q*_1_, *q*_2_; *q*_3_, *q*_4_) to (*q*_1_ − 1, *q*_2_ + 1; *q*_3_, *q*_4_), one of the *N_Ab_*(*t*) + *N_ab_*(*t*) lineages, which carry the *b* allele, must be chosen to recombine, and the rate is $q_{1}r\frac{N_{Ab}\left(t\right)+N_{ab}\left(t\right)-q_{2}-q_{4}}{2N_{t}}$. And the other transition rates can be obtained by similar rationale. Note that the selected allele first entered into the population at time *T*, so the states of the embedded Markov chain should satisfy *Q*(*T*) ∈ {(1, 0; *q*_3_, *q*_4_), (0, 1; *q*_3_, *q*_4_)} and *Q*(*t*) = (0, 0; *q*_3_, *q*_4_), *t* > *T*.

**Table 1:** Definitions of notations used in this article

Notation	Meaning
*n*_sample_	Total number of haplotypes in the sample
*n*	Number of selected haplotypes
*m*	Number of SNPs of a sample
*m_L_* and *m_R_*	Number of SNPs on the left and right sides of the mutant
D*_i_*_,_*_j_* = 0 or 1	The *j*th SNP of the *i*th haplotype
*N_t_*	Population size at time *t*
*T*	Allele age, or the time when the mutant arose in the population
*s*	The selection coefficient
*r*	Recombination fraction of the haplotype
*μ*	Mutation rate of the haplotype
*θ* = 4*Nμ*	The scaled mutation rate of the haplotype
*ρ* = 4*Nr*	The scaled recombination rate of the haplotype
*β_j_*	The proportion of ancestral haplotype region as a fraction of the length of the *j*th haplotype
ℋ = {*I_T_*, *I_T_*_−1_, …, *I*_1_, *I*_0_}	The allele frequency trajectory
*I_t_*	The number of the selected allele in the whole population at time *t*
*X_t_* = *I_t_*/(2*N_t_*)	The frequency of the selected allele at time *t*
*h*_1_ = (*R*_1_, *R*_2_, *M*_1_, …, *M_k_*)	A recoded haplotype which includes two recombination
	Coordinates and *k* mutation coordinates
T = {*h*_1_, …, *h_d_*}	The *d* different haplotypes of a sample
**n** = {*n*_1_, …, *n_d_*}	The number of haplotypes for each haplotype group in T
(T, **n**)*_t_*	The sample configuration at time *t*
*q*((T, **n**)*_t_*)	Sampling probability of the sample configuration (T, **n**) at time *t*
**e_j_** = (0, 0,…,1,…,0)	The *j*th unit vector
$\gamma \left(v,n\right)=\left(\begin{array}{c} n\\ 2 \end{array}\right){\left(\lambda_{v}X_{v}\right)}^{-1}+n\theta /2+n\rho /2$	The total rate for events at time *v*
λ*_t_* = *N_t_*/*N*_0_	The ratio of population size at *t* to that at the present
S*h_k_*	S denotes a shift operator, and S*h_k_* denotes deleting the first mutation coordinate of the *k*th haplotype
C*h_k_*	C denotes a coordinate change operator, and C*h_k_* denotes changing one of the two recombination coordinates of the *k*th haplotype and eliminating all mutation coordinates outside the ancestral regions delimited by the new recombination coordinates
R*_k_*T	The deleting operator that deletes *h_k_* haplotype from T
ℒ(*s*)	Likelihood function of the data
G^(^*^m^*^)^	The *m*th genealogical history, which consists of multiple steps of events, including recombination, mutation and coalescences
ℚ(ℋ)	Proposal distribution for ℋ in the importance sampling algorithm
ℚ(G|ℋ)	Proposal distribution for G conditional on a ℋ in the importance sampling algorithm

**Table 2:** Possible transitions from (*q*_1_, *q*_2_; *q*_3_, *q*_4_) and the rates for the two-locus haplotype model

Transition	Rate
(*q*_1_, *q*_2_; *q*_3_, *q*_4_)→(*q*_1_ − 1, *q*_2_; *q*_3_, *q*_4_)	$q_{1}\left(1-r\right)\frac{\left(q_{1}-1\right)}{N_{AB}\left(t\right)}$
(*q*_1_, *q*_2_; *q*_3_, *q*_4_)→(*q*_1_ − 1, *q*_2_ + 1; *q*_3_, *q*_4_)	$q_{1}r\frac{N_{Ab}\left(t\right)+N_{ab}\left(t\right)-q_{2}-q_{4}}{2N_{t}}$
(*q*_1_, *q*_2_; *q*_3_, *q*_4_)→(*q*_1_ −1, *q*_2_; *q*_3_ + 1, *q*_4_)	$q_{1}r\frac{N_{AB}\left(t\right)+N_{aB}\left(t\right)-q_{3}}{2N_{t}}$
(*q*_1_, *q*_2_; *q*_3_, *q*_4_)→(*q*_1_ −1, *q*_2_; *q*_3_, *q*_4_ + 1)	$q_{1}r\frac{N_{Ab}\left(t\right)+N_{ab}\left(t\right)-q_{2}-q_{4}}{2N_{t}}$

### A simplified multilocus model for haplotype structure

The model for two-locus haplotypes can be naturally extended to multilocus haplotypes. However, the extent of haplotype structure that is used to infer selection intensity and allele age usually spans a large region which covers several hundred kilobases, or even more than a mega-base. In such a large region, there may be hundreds of polymorphic sites. When the number of SNP loci increases, the number of possible states of the ancestral process increases exponentially, and the transition matrix becomes so large that numerical evaluation becomes impossible. It is thus necessary to develop a parsimonious model for multilocus haplotypes that is both computationally fast and statistically efficient.

The novel multilocus model we present here exploits the extents of the “ancestral haplotypes” retained during the selection process. We use the term “ancestral haplotype” to refer to the alleles at each SNP position on the ancestral chromosome, and the term “background haplotypes” to refer to the other haplotypes. In this model, we consider the interplay between selection and recombination acting upon the ancestral haplotype. As the selected allele increases its frequency, recombination breaks up the ancestral haplotypes and mixes them with the background haplotypes, resulting in the sample we observe at present. We make several simplifications or assumptions to expedite the computation in the sections to follow.

#### The ancestral state of each position along the haplotypes is assumed known:

For each position of a chromosome, it is assumed to be known whether the allele at that position is descended from an ancestral haplotype or one of the background haplotypes. In reality, the ancestral haplotype information cannot be observed directly from the data. The ancestral states and the break points of the ancestral haplotypes have to be inferred for each chromosome from patterns of SNP variation by other means (for example, the hidden Markov model for detecting recent positive selection, see Chen (2007)).

#### The haplotype structure of background haplotypes is ignored:

It is reasonable to believe that the primary information for the inference of allele age and selection intensity comes from the extent of the ancestral haplotypes retained during selection. For example, states of the jump process for a two-locus haplotype model are reduced to *Q*(*t*) = (*q*_1_, *q*_2_), and the absorbing states are now (1,0) and (0,1).

#### The population frequencies of ancestral haplotypes at time t are approximated using the expectations:

To obtain the transition rates in Table 2 for a two-locus model, the population counts of haplotypes *AB*, *Ab*, *aB*, and *ab* at time *t*, *N_AB_*(*t*), *N_Ab_*(*t*), *N_aB_*(*t*), and *N_ab_*(*t*), are needed. For a multilocus haplotype model, these values correspond to the population frequencies of haplotypes at time *t*. In studies of fine-scale disease mapping, these allele frequencies were assumed to be constant over time and identical to the observed frequencies in the current population (Rannala and Reeve 2001). When there is selection, the allele frequencies of nonrecombined haplotypes change over time, and their expectations have to be derived using a deterministic model. These expectations will be used as an estimate of the true haplotype frequencies at any time *t*. The details of the equations for the haplotype frequencies at time *t* can be seen in Appendix A.

#### Multiple “migrations” of ancestral haplotype fragments between selected and neutral haplotypes are ignored:

In the ancestral process of a two-locus haplotype model, there is a probability that a lineage of the neutral marker experiences two recombination events during the sweep process. In other words, the ancestral haplotype crosses over twice with a background haplotype at the marker position and the segment of the ancestral haplotype “migrates” to and then back from the group of neutral haplotypes. The probability for such events are small during a selective sweep, and thus are ignored (on order $O\left(\frac{1}{\text{log}{\left(\alpha \right)}^{2}}\right)$, where *α* = 2*N s*, see Etheridge *et al.* 2006).

Because of the assumptions (1)−(4), the state space of the ancestral process can be reduced by considering the unique pattern of ancestral haplotype lengths in combination with the occurrence of mutation since selection began. Because we have assumed that the ancestral states of SNPs along the chromosomes are known, for every haplotype, we can determine the break points of the ancestral haplotype caused by the recombination events nearest to the mutant, in addition to the locations where mutations occurred within each ancestral haplotype region. With this information known, the ancestral haplotype on each side of the mutant can be coded as follows: for every selected haplotype, we record the SNPs to the left and to the right sides that delimit the ancestral haplotype; if there are mutations within the ancestral haplotype regions, the positions of the mutants are also recorded and listed as “mutation coordinates” behind the two “recombination coordinates.” In Table 3, we give an example of 10 selected haplotypes consisting of 25 SNPs, among which the ancestral haplotype regions are highlighted. The selected mutant is located at position 18 (shown in boldface type). The left end of the ancestral haplotype for the first haplotype is 7 to the left of the mutant, and the right end is 6 to the right, such that the first haplotype is recorded as (7, 6). For haplotype 3, the full code is (12, 7, 21) with a mutation occurring in position 21. By this coding rule, the configuration of the sample listed in Column 3 of Table 3 is summarized in Column 3.

**Table 3:** An example of haplotype configuration to demonstrate the coding rules used to denote the haplotype structure

Haplotype Type	Count Number	Code
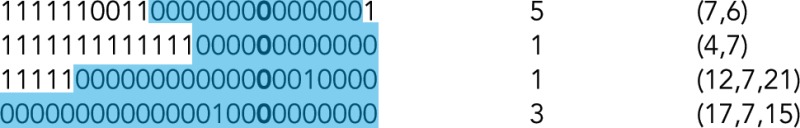		

There are 10 haplotypes with 25 single-nucleotide polymorphisms in four distinct groups in the sample. The mutant is located in position 18 and shown in boldface type. The ancestral region for each haplotype is highlighted. The codes for the four haplotype groups are listed in the third column

For a recoded haplotype type *h* = (*R*_1_, *R*_2_, *M*_1_, …, *M_k_*), the first two entries, corresponding to the left and right break points of the ancestral haplotypes, are the “recombination coordinates” and the other entries are the “mutation coordinates.” In this model, the transition among different haplotypes is caused by recombination and mutation. For the coded haplotypes consisting of only recombination coordinates, the transition among different haplotype types can occur only through recombination. If there are *m_L_* loci to the left of the mutant and *m_R_* loci to the right of the mutant, the total number of possible allele types is (*m_L_* + 1) × (*m_R_* + 1). The number of possible states is greatly reduced compared to a direct extension of the two-locus model, whose state space grows exponentially with number of SNPs.

In Table 4, we present a partial list of transition rates caused by recombination for a 4-locus haplotype model. Assume that the haplotype has 4 SNP loci, with the alleles on the ancestral haplotype being A, B, C, and D. A is the selected mutant, and the order of the four loci along the chromosome is the same as their alphabetical order. We use the notation [*ABCD*] to denote the intact segment of ancestral haplotype. And similarly [*AB* − *d*] indicates that the first two loci have the inherited ancestral haplotype of A and B, the allele of the third locus is arbitrary, and the fourth locus is a background haplotype. Examples in Table 4 show some of the one-step transition rates starting from state [*ABcd*]. For example, for haplotype [*ABcd*] to jump to [*ABCD*], one of the [*ABcd*] haplotypes should be chosen and recombination has to occur between allele B and c, in such a way that the chosen haplotype crosses over with haplotypes [*ABCD*], [*AbCD*] and [*a* − *CD*]. The one-step transition probability is: $r_{BC}\left\{P_{\left[ABCD\right]}\left(t\right)\cdot X_{t}+P_{\left[AbCD\right]}\left(t\right)\cdot X_{t}+P_{\left[a-CD\right]}\left(t\right)\cdot \left(1-X_{t}\right)\right\}$, with *X_t_* being the population allele frequency of allele *A* at time *t*, and *P*_[.]_(*t*) being the frequency of the haplotype in square parenthesis among either the selected haplotype or the background haplotype group, depending on the allele type carried by the particular haplotype at the selected mutant locus. Because of assumption (4) we made previously (also made by Durrett and Schweinsberg (2004)), the second and the third terms are small and can be ignored. With this simplification, the transition rate in Table 4 becomes *r_BC_* ⋅ *P*_[_*_ABCD_*_]_(*t*) ⋅ *X_t_*. Similarly, we can simplify the other transition rates shown in Table 4.

**Table 4:** The transition probabilities for some states of the multilocus haplotype model

Transition	Rate
[*ABcd*]→[*ABCD*]	$r_{BC}\left\{P_{\left[ABCD\right]}\left(t\right)\cdot X_{t}+P_{\left[AbCD\right]}\left(t\right)\cdot X_{t}+P_{\left[a-CD\right]}\left(t\right)\cdot \left(1-X_{t}\right)\right\}$
[*ABcd*]→[*ABCd*]	$r_{BC}\{P_{\left[ABCd\right]}(t)\cdot X_{t}+P_{\left[AbCd\right]}(t)\cdot X_{t}+P_{\left[a-Cd\right]}(t)\cdot (1-X_{t}))\}$
[*ABcd*]→[*Abcd*]	$r_{AB}\left\{P_{\left[Abcd\right]}\left(t\right)\cdot X_{t}+P_{\left[aBcd\right]}\left(t\right)\cdot \left(1-X_{t}\right)\right\}$

A mutation coordinate records the SNP position at which the haplotype has an allele mutated from the ancestral haplotype. We assume an infinitely-many-sites model for mutations on the haplotypes. According to Griffiths and Tavaré (1995), the set of nonrecombining haplotypes carrying these mutations is identical to a rooted gene tree, and the sequence of mutations corresponds to the path from the haplotype to the common ancestor [the root of the gene tree, see Griffiths and Tavaré (1995) for a detailed discussion]. We use the same notation scheme for mutations as Griffiths and Tavaré (1995). Because the haplotypes we investigate are from recombining regions, an additional constraint is added to reflect the effect of recombinations: the sequence of mutations we recorded as mutation coordinates includes only those located between the two recombination coordinates, that is, the subset of mutations on the retained ancestral haplotype region. For haplotype data from nonrecombining regions, the recombination coordinates are identical for all haplotypes (the left and right ends of the whole haplotype), and the mutation coordinates define a gene tree with the rooted genealogy, meaning that the state of the common ancestor of the sample is known (Griffiths and Tavaré, 1994b). This is the type of data analyzed by the approach of Coop and Griffiths (2004). Thus their method can be viewed as a special case of our method with no recombination.

### Sampling probability of a multilocus haplotype configuration

In the section *A simplified multilocus model for haplotype structure*, we described a novel simplified multilocus model that can dramatically reduce the state space of the haplotype ancestral process, and illustrated how to obtain the transition probabilities between different states. We now consider the computation of the probability of a sample of multilocus haplotypes.

A sample of selected haplotypes can be coded and summarized by the rules introduced in the section *A simplified multi-locus model for haplotype structure* and grouped into *d* distinct groups T = {*h_1_*, …, *h_d_*} with the corresponding multiplicities **n** = {*n*_1_, …, *n_d_*}. We define the sampling probability, *q*((T, **n**)*_t_*), to be the probability of observing the sample configuration (T, **n)** at *t* generations before the current generation. The entire history of the sample configuration {(T, **n**)*_t_*, *t* > 0} can be described by a Markov process that starts at time *t* = 0 and continues until reaching the absorbing state (T, **e_1_**) at a random time ***τ***, where **e_j_** denotes the unit vector **e_j_** = (0,0, …, 1, …, 0) with only the *j*th entry being 1. When *t* = 0, *q*((T, **n**)_0_) is the partial likelihood of the data which is sufficient for the inference of selection intensity and allele age. The sampling probability at time *t* can be obtained by recursively summing over all possible state paths in the backward Markov process. The recursive formula can be written as

$$q\left({\left(T,n\right)}_{t}\right)=\int_{t}^{\infty }\sum_{\left(T\prime^{,}n^{\prime }\right)}p\left({\left(T,n\right)}_{t}|{\left(T^{\prime },n^{\prime }\right)}_{v}\right)q\left({\left(T^{\prime },n^{\prime }\right)}_{v}\right)g\left(v|n,t\right)dv,$$ (2)

where $p\left({\left(T,n\right)}_{t}|{\left(T^{\prime },n^{\prime }\right)}_{v}\right)$ is the transition probability of jumping from state (T′, **n**′) at time *v* to state (T, **n**) at time *t*, and *g*(*v*|*n*, *t*) is the density function of the inter-arrival time to the next event given an event at time *t*.

As the Markovian ancestral process is restricted to the selected haplotypes, the process behaves as if in a population with temporally varying size {*I_t_*, *t* = 0.1, …, *T*}. If time is measured in a scale of 2*N*_0_ generations, the coalescent rate is $\left(\begin{array}{c} n\\ 2 \end{array}\right){\left(\lambda_{t}X_{t}\right)}^{-1}$, with *X_t_* = *I_t_*/2*N_t_* and λ*_t_* = *N_t_*/*N*_0_ being the population size ratio. With the same scaling, the mutation rate for the *i*th haplotype is *θ_i_* = 4*N*_0_*μβ_i_* = *θ β_i_*, and the recombination rate is *ρ_i_* = 4*N*_0_*cβ_i_* = *ρβ_i_*. *θ* = 4*N*_0_*μ* and *ρ* = 4*N*_0_*c* are the scaled mutation rate and recombination rate for the whole haplotype, and *β_i_* denotes the proportion of the retained ancestral haplotype region, or the inter-region between two recombination coordinates, out of the entire length of the *i*th haplotype. Note that *β_i_* changes over time with the change of recombination coordinates of the *i*th haplotype. The inter-arrival time to the next event, *v*, given that the last event happened at time *t* has a non-homogeneous exponential distribution, with the density function in the form of

$$g\left(v|n,t\right)=\gamma \left(v,n\right)\text{exp}\left(-\int_{t}^{v}\gamma (u,n)du\right),$$ (3)

*t* < *v* < ∞, where at time *v*, $\gamma \left(v,n\right)=\left(\begin{array}{c} n\\ 2 \end{array}\right){\left(\lambda_{t}X_{v}\right)}^{-1}+\sum_{i=1}^{n}\theta_{i}/2+\sum_{i=1}^{n}\rho_{i}/2$ is the rate for the any events. As the allele frequency trajectory {*X_t_*, 0 ≤ *t* ≤ *T*} is a discrete-time random process following the Wright-Fisher model, we adopt the geometric distribution for discrete time instead of using the continuous approximation in Equation 3:

$$g\left(v|n,t\right)=\gamma \left(v,n\right)\times \prod_{u=t+1}^{v-1}\left(1-\gamma \left(u,n\right)\right).$$ (4)

Conditional on an event happening at time *v*, the probabilities for the event being a mutation, recombination or coalescent are respectively

$$\frac{\sum_{i=1}^{n}\theta_{i}/2}{\gamma \left(v,n\right)},\;\frac{\sum_{i=1}^{n}\rho_{i}/2}{\gamma \left(v,n\right)}\;\mathrm{and}\;\frac{\left(\begin{array}{c} n\\ 2 \end{array}\right)\frac{1}{\lambda_{v}X_{v}}}{\gamma \left(v,n\right)}.$$ (5)

**Figure 3:**
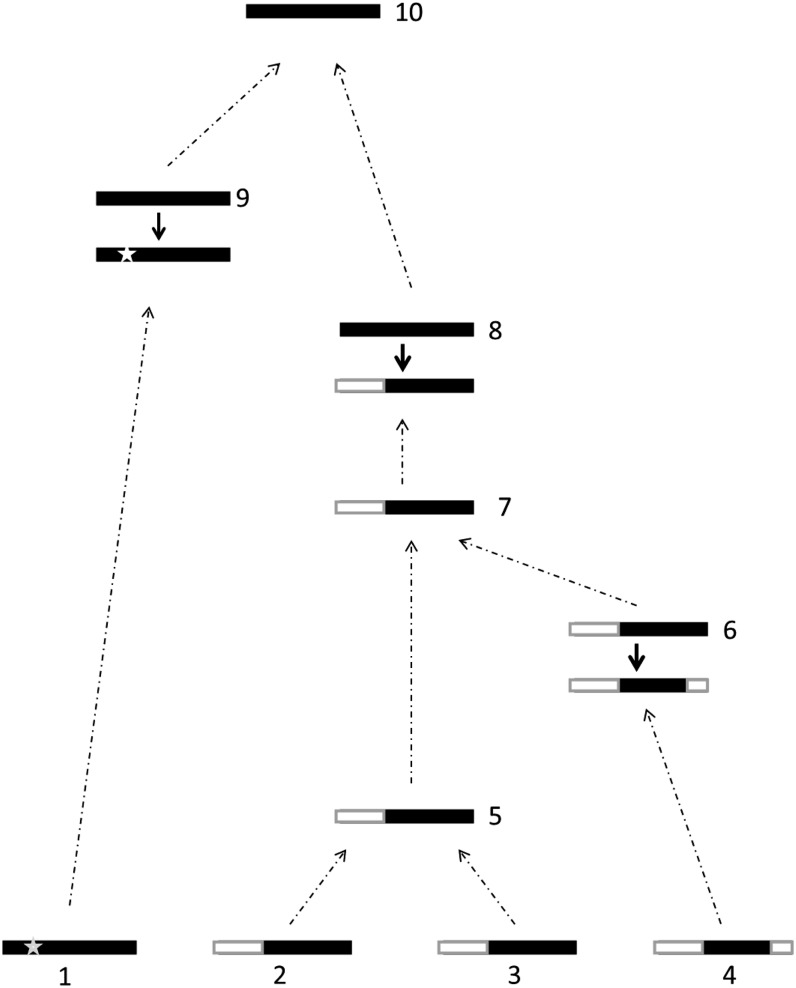
A realization of the genealogies for a sample of four haplotypes (lineages 1−4) to illustrate possible events in the genealogies. Black denotes the ancestral haplotype region (see the main text for the definition of “ancestral haplotypes”), and white denotes background haplotypes. A star denotes a neutral mutant arising on the ancestral haplotype. The present time, *t* = 0, is on the bottom. When going back in time, the events are coalescent (lineages 2 and 3 coalesce to the ancestral lineage 5), recombination (lineage 6 → lineage 4), coalescent (lineages 5 and 6 coalesce to the ancestral lineage 7), recombination (lineage 8 → lineage 7), mutation (on lineage 9), coalescent (lineages 8 and 9 coalesce to lineage 10) in sequence.

If a mutation occurs, one of the lineages in the sample is chosen to mutate into other types according to the mutation model, and the mutation coordinate of that haplotype is modified correspondingly; if a coalescence event occurs within the *j*th haplotype group, two of the existing lineages with haplotype *h_j_* are chosen at random to coalesce, and the number of lineages in the *j*th haplotype group, *n_j_*, is decreased by 1; otherwise, a position along the haplotype is chosen for the recombination event to occur with the consequence that one of the recombination coordinates is changed to record the recombination at that position (see Figure 3 for a realization of the genealogical history for a sample of four haplotypes).

We now present the detailed recursion equation (Equation 6), expressed as a sum over the above three types of events in a way corresponding to Equation 2 for our model. Under the infinitely-many-sites mutation model (Watterson 1975) and the proposed multilocus haplotype model for the extent of ancestral haplotypes, summing over possible one-step configuration changes at time *v* leads to the following equation:

$$\begin{array}{c} \ell_{q}\left({\left(T,n\right)}_{\text{v}}\right)=\sum_{k:n_{k}\geq 2}\frac{n\left(n_{k}-1\right)\frac{1}{\lambda_{v}X_{v}}}{2\gamma \left(v,n\right)}q\left({\left(T,n-e_{k}\right)}_{\text{v}}\right)\\ +\sum_{\begin{array}{c} k:n_{k}=1,h_{k}\;\text{distinct},\\ Sh_{k}\neq h_{j}\;\text{for}\;\text{all}\;j \end{array}}\frac{\theta \beta_{k}}{2\gamma \left(v,n\right)}q\left({\left(S_{k}T,n\right)}_{\text{v}}\right)\\ +\sum_{k:n_{k}=1,h_{k}\;\text{distinct}\;}\sum_{j:Sh_{k}=h_{j}}\frac{\theta \left(n_{j}+1\right)\beta_{j}}{2\gamma \left(v,n\right)}\\ \times q\left({\left(R_{k}T,R_{k}\left(n+e_{j}\right)\right)}_{\text{v}}\right)\\ +\sum_{i}\sum_{j:Ch_{i}=h_{j},n_{j}\geq 0,i\neq j}\frac{\beta_{j}\rho }{2\gamma \left(v,n\right)}\left(n_{j}+1\right)p_{h_{j},h_{i}}\\ \times q\left({\left(Ch_{i}T,n-e_{i}+e_{j}\right)}_{v}\right). \end{array}$$ (6)

The notation in Equation 6 has the following meaning: **e_k_** is the *k*th unit vector, representing a multiplicity of the *k*th distinct haplotype; *β_l_* is the length of ancestral haplotype region divided by length of the *l*th haplotype. We follow the notation of Griffiths and Tavaré (1995) and use several operators to denote the changes of sample configuration: S is the shift operator that can be operated on a specific haplotype *h_k_* or the entire haplotype set T. Specifically, S*h_k_* represents the haplotype obtained by deleting the first mutation coordinate of haplotype *h_k_*. Similarly, S*_k_*T represents the new set of distinct haplotypes obtained by deleting the first mutation coordinate of the *k*th distinct haplotype, *h_k_*, in T. Another operator that operates on the entire haplotype is R*_k_* which removes the *k*th distinct haplotype *h_k_* from the set T. The third coordinate change operator C is defined in this manuscript to denote the coordinate changes caused by recombinations: C*h_i_* changes one of the two recombination coordinates of haplotype *i*, $R_{h_{i}}=\left\{R_{h_{i},1},R_{h_{i},2}\right\}$, and eliminates all mutation coordinates outside the regions delimited by $R_{h_{j},1}$ and $R_{h_{j},2}$.

Next we explain how we derive the recursive formula in Equation 6. Starting from time *v* back from the present, there are four possible paths to arrive at the sample configuration (T, **n**) at time *v*: (1) a coalescent event occurred at time *v*, and a possible sample configuration prior to time *v* was (T, **n** − **e_k_**); (2) a mutation occurred on a haplotype *h_k_* that had only single multiplicity, or *n_k_* = 1, at time *v*, and a new mutation coordinate was added to *h_k_*; (3) a mutation occurred on a haplotype *h_j_* that had multiplicity greater than 1, or *n_j_* > 1, at time *v*, and a new haplotype *h_k_* with *n_k_* = 1 was generated by adding the new mutation coordinate to *h_j_*; (4) a recombination event occurred, and altered one of the recombination coordinates of *h_j_* by that of *h_i_*. Note that a recombination event not only changes the recombination coordinate, it also changes the mutation coordinates: after the recombination coordinates are changed by a recombination event, all the mutation coordinates of that haplotype are checked, and only those located within the interregion between the two new recombination coordinates are kept. The four terms on the RHS of Equation 6 correspond to the above four paths respectively, and the derivation of the first three terms follows Griffiths and Tavaré (1994a, 1995). In the first path, the sample configuration at time *v* compatible with the occurrence of coalescence is (T, **n** − **n_k_**), the probability that the event occurred at time *v* is a coalescent event is $\frac{\left(\begin{array}{c} n\\ 2 \end{array}\right)}{\lambda_{v}X_{v}\gamma \left(v,n\right)}$. And whenstarting from the configuration (T, **n** − **n_k_**) and going forward in time, the probability that one of the *n_k_* − 1 haplotype **e***_k_* is chosen to duplicate is $\frac{n_{k}-1}{n-1}$ (Griffiths and Tavaré, 1994a). The one-step transition probability of *p*((T, **n**)_v_|(T, **n** − **e_k_**)_v_) is then $\frac{n_{k}-1}{n-1}\frac{\left(\begin{array}{c} n\\ 2 \end{array}\right)}{\lambda_{v}X_{v}\gamma \left(v,n\right)}$. Note that a restriction for haplotype group *h_k_* is that there must be more than one lineage in group *h_k_* at time *v*. Summing over all possible haplotype groups that have multiplicity *n_k_* ≥ 2 at time *v*, and are compatible for coalescent events to occur, we obtain the first term of Equation 6. The second and the third path correspond to the cases when the event occurring at time *v* is a mutation. Under the assumption of the infinitely many-sites model, if a mutation event occurs, it can result only in one of the single-multiplicity haplotype groups at time *v* (*n_k_* = 1) and the mutation coordinate must be a singleton in the sample configuration at time *v*. In both the second and the third paths, the chance that a mutation occurred at time *v* is $\frac{\sum_{l=1}^{n}\theta_{l}}{2\gamma \left(v,n\right)}$. The configuration at time *v* compatible with the occurrence of second path is (S*_k_*T, **n**), and the probability for the mutation to happen to haplotype *h_k_* is $\frac{\beta_{k}}{\sum_{l=1}^{n}\beta_{l}}$. Summing over all haplotypes satisfying *n_k_* = 1 and S*h* ≠ *h_j_* for all *j* yields the second term in Equation 6. In the third path, the probability for the mutation to happen to haplotype *h_k_* is $\sum_{j:Sh_{k}=h_{j}}\frac{\left(n_{j}+1\right)\beta_{j}}{\sum_{l=1}^{n}\beta_{l}}$ with the sample configuration prior to the event being $\left(R_{k}T,R_{k}\left(n+e_{j}\right)\right)$ for all k with *n_k_* = 1 at time *v*. In the fourth path, recombination occurs with a probability of $\frac{\sum_{l=1}^{n}\beta_{l}\rho /2}{\gamma \left(v,n\right)}$. If recombination causes a haplotype *h_j_* to become *h_i_*, the haplotype configuration prior to the event is (C*h_i_*T, (**n** − **e_i_** + **e_j_**)) and the probability for a haplotype *h_j_* changing into *h_i_* is $\frac{\beta_{j}\left(n_{j}+1\right)}{\sum_{l=1}^{n}\beta_{l}}p_{h_{j},h_{i}}$, with *n_j_* ≥ 0. The transition probability $p_{h_{j},h_{i}}$ between different haplotypes follows the multilocus haplotype model presented in the section *A simplified multi-locus model for haplotype structure*, where *h_i_* and *h_j_* correspond to one of the distinct haplotypes defined by the “recombination coordinates”. Combining these possibilities and averaging over the time to the first event more ancient than *t*, the sampling distribution of haplotype configuration, (T, **n**), is analogous to Equation 2:

$$q\left({\left(T,n\right)}_{t}\right)=\int_{t}^{\infty }\ell_{q}\left({\left(T,n\right)}_{v}\right)\gamma \left({\left(T,n\right)}_{v}\right)\text{exp}\left(-\int_{t}^{v}\gamma \left({\left(T,n\right)}_{u}\right)du\right)dv$$ (7)

Although we have reduced the state numbers defined by recombinations from $2^{\left(m_{L}+m_{R}+1\right)}$ to (*m_L_* + 1) × (*m_R_* + 1) for the simplified multilocus haplotype model, it is still difficult to numerically solve the distribution function induced by the Markov process. Therefore ,we still need to use the importance sampling algorithms to estimate the sampling probability, as will be shown in following sections.

## Importance sampling and proposal distributions

### Likelihood and importance sampling

Importance sampling algorithms are used to efficiently sample from the probability spaces of frequency trajectories and intra-allelic genealogies in order to approximate the integral in the likelihood function (see Equation 1). The likelihood function in Equation 1 can be expressed as

$$ℒ\left(s\right)={\int \int \mathbb{P}\left(D|G\right)\frac{\mathbb{P}\left(G|ℋ\right)}{\mathbb{Q}\left(G|ℋ\right)}\mathbb{Q}\left(G|ℋ\right)\frac{\mathbb{P}\left(ℋ|s,\Gamma \right)}{\mathbb{Q}\left(ℋ|s,\Gamma \right)}\mathbb{Q}\left(ℋ|s,\Gamma \right)dGdℋ}^{},$$ (8)

where ℚ(G|ℋ) and ℚ(ℋ|*s*, Γ) are the proposal distributions that have non-zero weight only on genealogies and trajectories compatible with the data D (that is, ℙ(D| G) = 1). Suppose that *M* random frequency trajectories and *L* genealogical histories for each of the trajectories are sampled, then the approximation to Equation 8 becomes

$$ℒ\left(s\right)\approx \frac{1}{M}\frac{1}{K}\sum_{m=1}^{M}\sum_{k=1}^{K}\frac{\mathbb{P}\left({ℋ}^{\left(m\right)}|s,\Gamma \right)}{\mathbb{Q}\left({ℋ}^{\left(m\right)}|s,\Gamma \right)}\frac{\mathbb{P}\left(G^{\left(k\right)}|{ℋ}^{\left(m\right)}\right)}{\mathbb{Q}\left(G^{\left(k\right)}|{ℋ}^{\left(m\right)}\right)},$$ (9)

where ℋ^(^*^m^*^)^ and G^(^*^k^*^)^ are the *m*th and *k*th independent samples from the proposal distributions. The ratio $\frac{\mathbb{P}\left(\cdot \right)}{\mathbb{Q}\left(\cdot \right)}$ is called the importance weight. The importance sampling algorithm for the genealogies will be presented in the section *A proposal distribution for sampling genealogical histories conditional on a trajectory* and the algorithm for the allele frequency trajectories in the section *The proposal distribution for sampling allele frequency trajectories of the selected allele in a population of time-varying size*. We illustrate the proposal distributions and the calculation of importance weights in those two sections.

Allele age, *T*, is not explicitly expressed as a variable in the likelihood function. It is the end point of the frequency trajectory, and thus depends on *s* through ℙ(ℋ|*s*). Once the maximum likelihood estimate $\hat{s}$ is found, the posterior distribution of *T* can be obtained from the repeated samples of ℋ given $s=\hat{s}$. This method for estimating allele age has been used by Coop and Griffiths (2004), Saunders *et al.* (2005) and Wood *et al.* (2005), while it is different from the Bayesian approach of Slatkin (2008), who assumed a prior for allele age and jointly inferred both selection intensity and allele age.

### A proposal distribution for sampling genealogical histories conditional on a trajectory

The recursion of the genealogical histories given in Equation 6 for the likelihood of the data cannot be computed exactly for large data sets since there are too many compatible sets of ancestral states. We adopt an importance sampling algorithm to approximate the likelihood by Monte Carlo methods. There are many ways of constructing the proposal distributions for the importance sampling algorithm (Griffiths and Tavaré, 1994b; Stephens *et al.* 2001; Paul *et al.* 2011). Here we follow the scheme developed by Griffiths and Tavaré (1994b). As described in previous sections, the infinitely-many-sites model for mutations in conjunction with the simplified multi-locus haplotype model is assumed.

In the algorithm, a Markov process starting from the configuration in the current generation (T, **n**)_0_, conditional on a randomly sampled historical frequency trajectory {*X_t_*, *t* = 0, …, *T*}, is constructed and simulated backward in time until reaching the absorbing state (T, **e_1_**)*_τ_* at time ***τ***. The algorithm is summarized as follows:

1. Generate time to the next event, *v*, by the density function given in Equation 4;
2. Choose one of the three possible events (recombination, mutation or coalescence) from the proposal distribution. We first define the total rate that any event occurs at time v as
  $$\hbar \left({\left(T,n\right)}_{v}\right)=\sum_{k,n_{k}\geq 2}n\left(n_{k}-1\right)\frac{1}{\lambda_{v}X_{v}}+\sum_{i}\sum_{\begin{array}{c} j:Ch_{i}=h_{j}\\ n_{j}\geq 0,i\neq j \end{array}}\beta_{j}\rho \left(n_{j}+1\right)p_{h_{j},h_{i}}+\theta m,$$ (10)
  where
  $$m=\sum_{k,n_{k}=1,h_{k}\;\text{distinct},Sh_{k}\neq h_{j}\text{for all j}}\beta_{k}+\sum_{k:n_{k}=1,h_{k}\text{distinct}}\sum_{j:Sh_{k}=h_{j}}\left(n_{j}+1\right)\cdot \beta_{j}.$$ (11)
  The **proposal distribution** is designed in such a way that a possible event at time *v* is chosen with probability in proportion to the size of each term in *ℏ*((T, **n**)*_v_*):
  $$p\left({\left(T^{\prime },n^{\prime }\right)}_{v}|{\left(T,n\right)}_{v}\right)=\{\begin{array}{cc} \frac{n\left(n_{k}-1\right)\frac{1}{\lambda_{v}X_{v}}}{\hbar \left({\left(T,n\right)}_{v}\right)}, & \left(T^{\prime },n^{\prime }\right)=\left(T,n-e_{k}\right)\;\mathrm{and}\;n_{k}\geq 2,\\ \frac{\theta \beta_{k}}{\hbar \left({\left(T,n\right)}_{v}\right)}, & \left(T^{\prime },n^{\prime }\right)=\left(S_{k}T,n\right),\\ \frac{\theta \beta_{j}\left(n_{j}+1\right)}{\hbar \left({\left(T,n\right)}_{v}\right)}, & \left(T^{\prime },n^{\prime }\right)=\left(R_{k}T,R_{k}n+e_{j}\right),\\ \frac{\beta_{j}\rho \left(n_{j}+1\right)p_{h_{j},h_{i}}}{\hbar \left({\left(T,n\right)}_{v}\right)}, & \left(T^{\prime },n^{\prime }\right)=\left(Ch_{i}T,n-e_{i}+e_{j}\right). \end{array}$$ (12)
3. Update the configuration to reflect the chosen event. Let G*_j_*, *j* ≥ 0 denote the *j*th event during the genealogical history, and G_0_ = (T, **n**)_0_. ℚ(G_j_ |G*_j_*_−1_) is the transition probability of the backward Markov process determined by the proposal distribution (Equation 12). Similarly, ℙ(G*_j_*_−1_ |G*_j_*) is the transition probability of the forward Markov process. The sequential importance weight for the *j*th step change $\frac{\mathbb{P}\left(G_{j-1}|G_{j}\right)}{\mathbb{Q}\left(G_{j}|G_{j-1}\right)}$ is estimated. Here we illustrate the calculation of the importance weight for the case in which the chosen event is G*_k_* = (T, **n** − **e*_k_***). As shown in the section *Sampling probability of a multilocus haplotype configuration*, $\mathbb{P}\left(G_{j-1}|G_{j}\right)=\frac{\left(\begin{array}{c} n\\ 2 \end{array}\right)\left(n_{k}-1\right)}{\lambda_{v}X_{v}\gamma \left(v,n\right)\left(n-1\right)}$, and from Equation 12 we have $\mathbb{Q}\left(G_{j}|G_{j-1}\right)=\frac{n\left(n_{k}-1\right)\frac{1}{\lambda_{v}X_{v}}}{\hbar \left({\left(T,n\right)}_{v}\right)}$. Taking the ratio of the two terms, we obtain the importance weight for the *j*th step: $\frac{\hbar \left({\left(T,n\right)}_{v}\right)}{2\gamma \left(v,n\right)}$. Table 5 provides more details of importance weights for other events.
4. Repeat steps 1−3 to continue generating the historical events in the genealogy backward in time.
5. Stop when the absorbing state is reached, that is, a single lineage remains in the sample configuration, (T, **e**_1_), or if the proposed time for next event is beyond the end of the frequency trajectories;
6. Assume that there are *I* steps until the Markov chain reaches the absorbing states, the ratio of the forward/backward paths is the product of sequential importance weights:
  $$\frac{\mathbb{P}\left(G^{\left(k\right)}|ℋ\right)}{\mathbb{Q}\left(G^{\left(k\right)}|ℋ\right)}=\prod_{j=1}^{l}\left[\frac{\mathbb{P}\left(G_{j-1}|G_{j}\right)}{\mathbb{Q}\left(G_{j}|G_{j-1}\right)}\right]\cdot q\left({\left(T,e_{1}\right)}_{\tau }\right),$$ (13)
  and is used in the likelihood function. For those paths with time beyond the end of the given frequency trajectories, {*X_t_*}, the ratio is set to zero, which means the sample is rejected.

**Table 5:** The proposal distribution and importance weights for the importance sampling algorithm presented in the section *A proposal distribution for sampling genealogical histories conditional on a trajectory*

G*_j_*	ℚ(G*_j_*|G*_j_*_−1_)	ℙ(G*_j_*_−1_|G*_j_*)	Importance Weight
(T, **n** − **e_k_**)	$\frac{n\left(n_{k}-1\right)}{\lambda_{v}X_{v}\hbar \left({\left(T,n\right)}_{v}\right)}$	$\frac{\left(\begin{array}{c} n\\ 2 \end{array}\right)\left(n_{k}-1\right)}{\lambda_{v}X_{v}\gamma \left(v,n\right)\left(n-1\right)}$	$\frac{\hbar \left({\left(T,n\right)}_{v}\right)}{2\gamma \left(v,n\right)}$
(S*_k_*T, **n**)	$\frac{\theta \beta_{k}}{\hbar \left({\left(T,n\right)}_{v}\right)}$	$\frac{\sum_{l=1}^{n}\beta_{l}\theta /2}{\gamma \left(v,n\right)}\frac{\beta_{k}}{\sum_{l=1}^{n}\beta_{l}}$	$\frac{\hbar \left({\left(T,n\right)}_{v}\right)}{2\gamma \left(v,n\right)}$
(R*_k_*T, R*_k_*(**n** + **e_j_**))	$\frac{\theta \left(n_{j}+1\right)\beta_{j}}{\hbar \left({\left(T,n\right)}_{v}\right)}$	$\frac{\sum_{l=1}^{n}\beta_{l}\theta /2}{\gamma \left(v,n\right)}\frac{\left(n_{j}+1\right)\beta_{j}}{\sum_{l=1}^{n}\beta_{l}}$	$\frac{\hbar \left({\left(T,n\right)}_{v}\right)}{2\gamma \left(v,n\right)}$
(C*h_i_*T, **n** − **e_i_** + **e_j_**)	$\frac{\beta_{j}\rho \left(n_{j}+1\right)p_{h_{j},h_{i}}}{\hbar \left({\left(T,n\right)}_{v}\right)}$	$\frac{\beta_{j}\rho \left(n_{j}+1\right)p_{h_{j},h_{i}}}{\gamma \left(v,n\right)}$	$\frac{\hbar \left({\left(T,n\right)}_{v}\right)}{2\gamma \left(v,n\right)}$

{G} denotes four possible events of the genealogical history. ℙ(G*_j_*_−1_|G*_j_*) and ℚ(G*_j_*|G*_j_*_−1_) are the one-step transition probability of the forward and backward Markov process constructed for simulating the genealogical history. The importance weight is estimated by $\frac{\mathbb{P}\left(G_{j-1}|G_{j}\right)}{\mathbb{Q}\left(G_{j}|G_{j-1}\right)}$

### The proposal distribution for sampling allele frequency trajectories of the selected allele in a population of time-varying size

We use the backward Wright-Fisher model under selection to sample the allele frequency trajectories. The importance sampling algorithm for sampling frequency trajectories of the selected allele is described as follows. A detailed explanation can be found in the original paper (Slatkin 2001).

1. Given a selection intensity s and parameter set Γ, a sample path is simulated from *t* = 0 (current generation) with *I*_0_ copies of A, and then proceeds backward from generation to generation assuming the following binomial distribution:
  $$\mathbb{P}\left(I_{t}|I_{t-1}\right)=\left(\begin{array}{c} 2N_{t}\\ I_{t} \end{array}\right)Y_{t-1}^{\prime I_{t}}{\left(1-Y_{t-1}^{\prime }\right)}^{2N_{t}-I_{t}},$$ (14)
  where $Y_{t-1}^{\prime }$ satisfies: $Y_{t-1}^{\prime }\frac{1+s_{1}Y_{t-1}^{\prime }+s_{2}\left(1-Y_{t-1}^{\prime }\right)}{1+s_{1}Y_{t-1}^{\prime 2}+2s_{2}Y_{t-1}^{\prime }\left(1-Y_{t-1}^{\prime }\right)}=Y_{t-1}$, and *Y_t_*_−1_ = *I_t_*_−1_/(2*N_t_*_−1_). The backward process is stopped at time *T* when the allele is lost. The probability of the backward process is calculated as: ${\mathbb{P}}_{B}\left({ℋ}^{\left(m\right)}\right)=\prod_{t=1}^{T}\mathbb{P}\left(I_{t}|I_{t-1}\right).$
2. For a frequency trajectory ℋ^(^*^m^*^)^ simulated in Step 1, the probability it is generated by the forward process is computed. In the Wright-Fisher model with selection, the number of allele A from generation *t* to generation *t* − 1 follows a binomial distribution:
  $$\mathbb{P}\left(I_{t-1}|I_{t}\right)=\left(\begin{array}{c} 2N_{t-1}\\ I_{t-1} \end{array}\right)X_{t}^{\prime I_{t-1}}{\left(1-X_{t}^{\prime }\right)}^{2N_{t-1}-I_{t-1}},$$ (15)
  with
  $$X_{t}^{\prime }=X_{t}\frac{1+s_{1}X_{t}+s_{2}\left(1-X_{t}\right)}{1+s_{1}X_{t}^{2}+2s_{2}X_{t}\left(1-X_{t}\right)},$$ (16)
  which is the allele frequency of A after selection in generation *t*. In Equation 16, *X_t_* = *I_t_*/(2*N_t_*) is the frequency of allele A before selection in generation *t*. The probability of the sample path ℋ^(^*^m^*^)^ is
  $${\mathbb{P}}_{F}\left({ℋ}^{\left(m\right)}\right)=\prod_{t=T}^{1}\mathbb{P}\left(I_{t-1}|I_{t}\right),$$ (17)
  where ℙ(*I_T_*_−1_|*I_T_*) = 1 if *I_T_*_−1_ = 1 and 0 otherwise. And the subscript *F* indicates that the process is “forward” in time.
3. The importance weight is calculated as (Slatkin 2001):
  $$\frac{\mathbb{P}\left({ℋ}^{\left(m\right)}\right)}{\mathbb{Q}\left({ℋ}^{\left(m\right)}\right)}=\frac{{\mathbb{P}}_{F}\left({ℋ}^{\left(m\right)}\right)}{{\mathbb{P}}_{B}\left({ℋ}^{\left(m\right)}\right)}\dot{N},$$ (18)
  where $\dot{N}$ is the population size at the first generation after the allele is lost in the backward process. The multiplication of $\dot{N}$ is needed in Equation 18, since the rate of influx of new mutations is proportional to the population size of that generation.

## Applications

### Simulation

**Figure 4:**
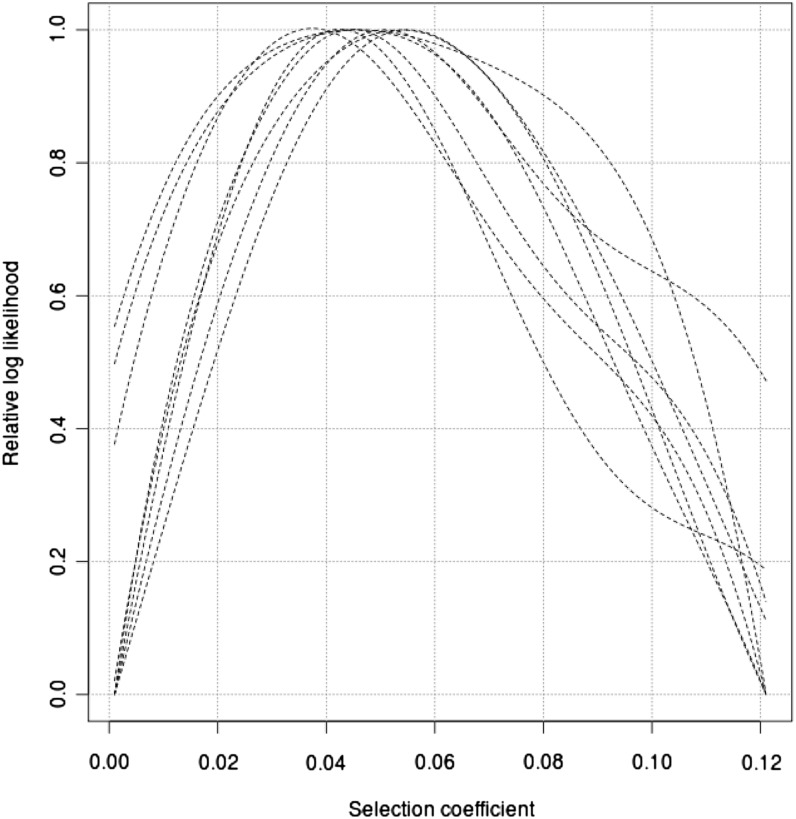
The relative likelihood curve for the simulated data with the selection coefficient *s* = 0.05 and a constant population size *N* = 10,000. The comparison of eight estimates of the likelihood curves is presented. Each estimate is an independent run of our method on different simulated data. The results are from 1 million iterations.

**Figure 5:**
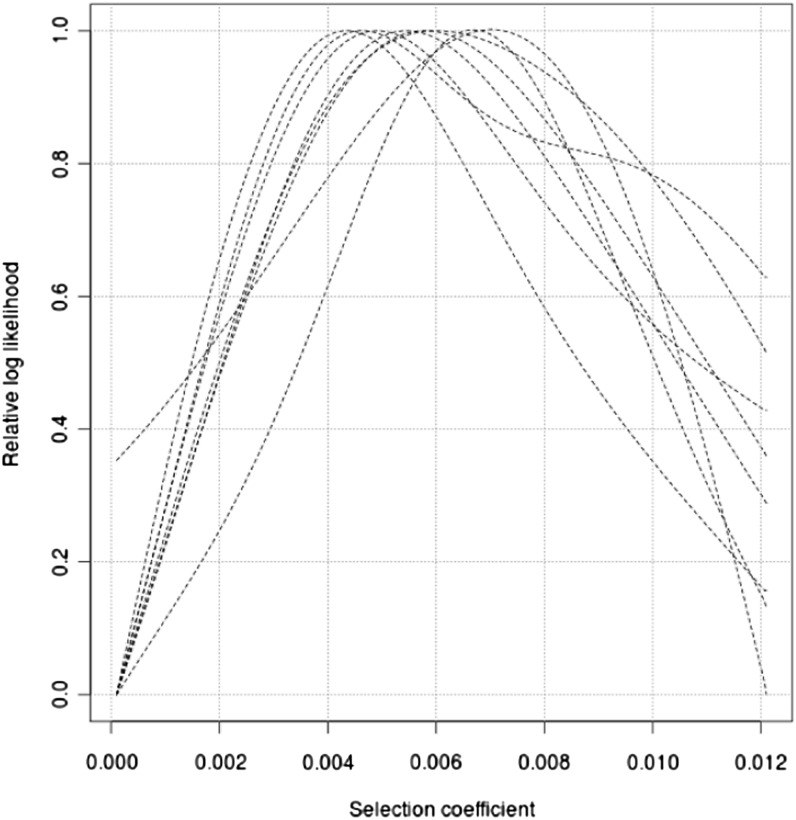
The relative likelihood curve for the simulated data with the selection coefficient *s* = 0.005 and a constant population size *N* = 10,000. The comparison of eight estimates of the likelihood curves is presented. Each estimate is an independent run of our method on different simulated data. The results are from 1 million iterations.

Using the coalescent simulator SelSim (Spencer and Coop 2004), data are generated for two sets of parameters corresponding to medium and strong selection respectively: *θ* = 4*Nμ* = 500, *ρ* = 4*Nr* = 500, *Ns* = 50, and *θ* = 4*Nμ* = 500, *ρ* = 4*Nr* = 500, *Ns* = 500, where *θ*, *ρ*, and *Ns* represent the mutation rate, recombination rate and selection coefficient scaled by the effective population size. The frequencies of the selected alleles at the present are chosen to be 0.60. Since the Moran model is used in SelSim, whereas the Wright-Fisher model is used in our method, the effective population size in the simulations is scaled to match that of a Wright-Fisher model by multiplying by a factor of 2 (Watterson 1975). We estimate the log-likelihood of *s* for a range of selection coefficients with the other parameters in Γ known, assuming that the population has a constant size of 10,000. The curves of the log-likelihood over the grid of *s* values are smoothed by a local polynomial smoother. This smoother fits a linear function to a subset of data points within a local window of the target point where the log-likelihood is to be estimated. The fitting is carried out by the weighted least square regression, which gives more weight to points close to the target point and less weight to distant points. The log-likelihood is thus estimated as the fitted value at the target point. The size of the local window or the bandwidth is chosen by eye for each curve. The log-likelihood curves are plotted in Figure 4 and Figure 5.

To evaluate the performance of the importance sampling approximation, we perform eight independent simulations for every parameter combination. One million iterations in the importance sampling algorithm are required to ensure good estimates, and the likelihood curves are presented together in Figure 4 and Figure 5. For the data set simulated with *s* = 0.005, the MLE ranges from 0.0041 to 0.0073. For the data set with *s* = 0.05, the MLE ranges from 0.032 to 0.0543.

### Glucose-6-phosphate dehydrogenase (*G6PD)*

The *G6PD* gene is located on the X-chromosome. Some alleles are known to confer the resistance to malaria (Ruwende *et al.* 1995). Case-control studies have demonstrated that a common variant, *G6PD-202A*, reduces the risk of malaria by approximately 50% (Ruwende *et al.* 1995). This allele is at low frequency in most populations but has an intermediate frequency in sub-Saharan Africa. Several population genetic studies have investigated the effect of a recent selective sweep in this region (Tishkoff *et al.* 2001; Sabeti *et al.* 2002). Here we use the data in Sabeti *et al.* (2002), which consists of 252 males from there African populations in a 440-Kb region covering the *G6PD* gene. We analyze only the 60 haplotypes from the Beni population. There are 10 haplotypes containing the 202-A allele in the sample. We assume that the frequency of the selected allele in the Beni population is the same as that estimated from the sample which is 0.1667. The recombination fractions among SNPs are obtained by interpolation with the Oxford fine-scale recombination map (Myers *et al.* 2005). The recombination rate in the *G6PD* gene region is heterogeneous with two recombination hot-spots, and the overall averaged recombination rate for the region is 1.4410 cM/Mb. We determine the end points of ancestral haplotypes and mutations by running the hidden Markov model (Chen 2007). The data configuration is coded by the rules presented in the section *A simplified multilocus model for haplotype structure* as shown in Table 6.

**Table 6:** The sample configuration of the *G6PD* data according to coding rules in the section *A simplified multilocus model for haplotype structure*

Haplotype Type	Count Number
(11, 7)	5
(4, 7)	1
(12, 7)	1
(17, 7)	3

**Figure 6:**
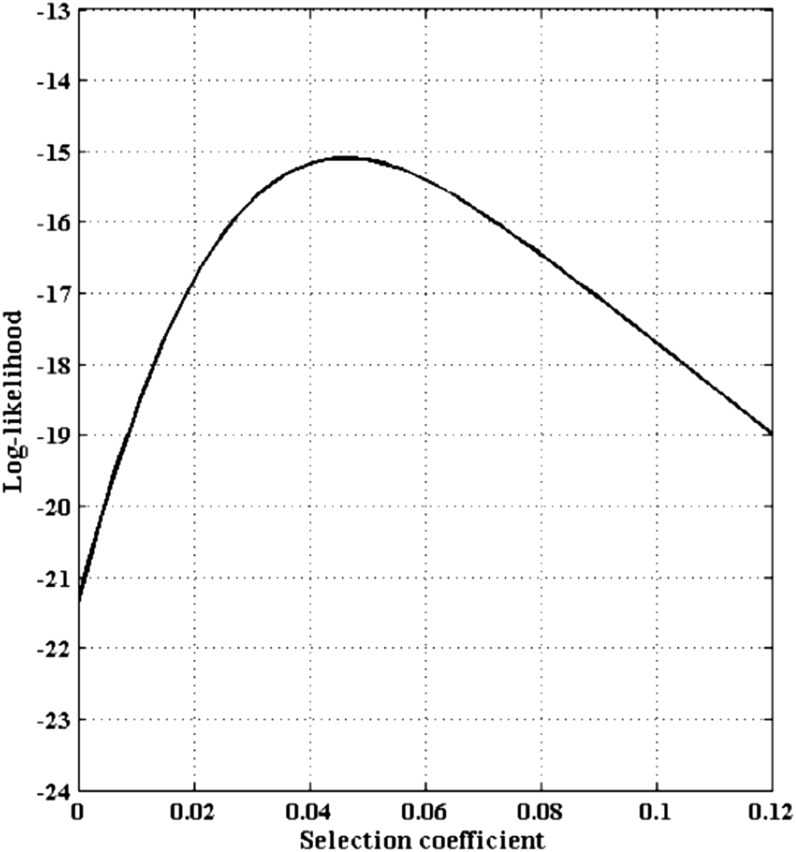
Likelihood curve for the *G6PD* data as a function of the selection coefficient with a constant population size of 10,000. The likelihood curve is smoothed by a local polynomial smoother. The point estimate of the selection coefficients is 0.0456 with the 95% confidence interval of (0.0144, 0.0769).

**Figure 7:**
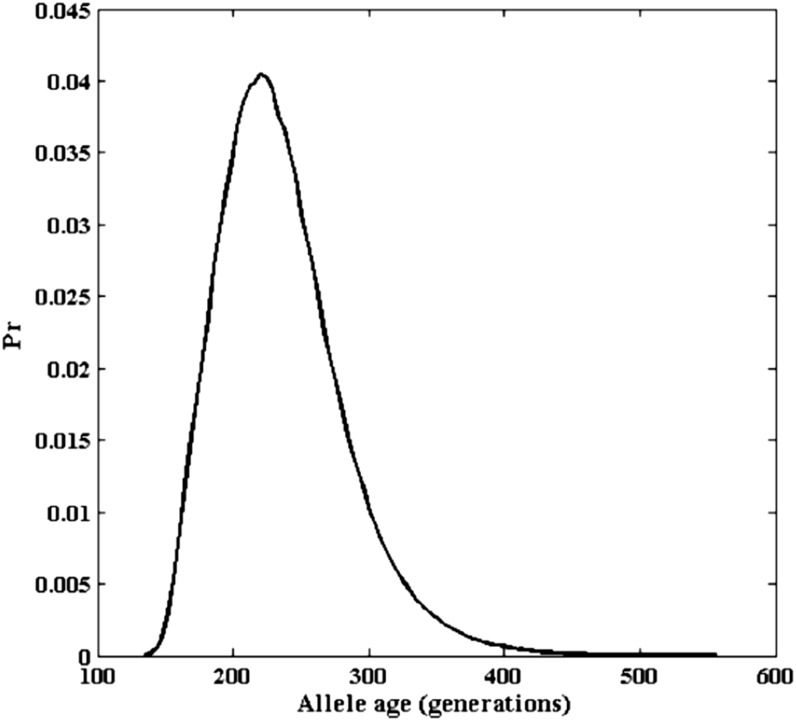
The posterior distribution of the allele age in generations when the selection coefficient is set to the value estimated from Figure 6.

Because the hidden Markov model analysis indicates there are no mutations in the ancestral haplotype regions, we set *θ* to 0.0. We assume an effective population size of *N* = 10,000, which is constant over time. Because *G6PD* is X-linked, *N_e_* is 3/4 of the autosomal size. We assume an additive model for selection, which means the fitness’s of the three genotypes aa, Aa, and AA are 1, 1 + 1/2*s*, and 1 + *s*, respectively. The likelihood of the selection coefficient is estimated by our method from 1 million iterations of the importance sampling algorithm. The log-likelihood curve is plotted in Figure 6. The selection coefficient is estimated to be 0.0456 (95% confidence interval of 0.0144−0.0769). From the estimated selection coefficient, the age of the 202-A allele can be estimated. As shown in Figure 7, given the selection coefficients estimated, the corresponding posterior distribution of allele age is plotted.

## Discussion

We have developed a likelihood method for estimating selection intensity and allele age from haplotype structure of multilocus SNPs closely linked to a selected mutant. The likelihood is based on the proposed simplified multilocus haplotype model, which describes the ancestral process of haplotype extent under the joint effects of selection, recombination and mutation. In this model, the state space of the ancestral process is determined by the extent of intact ancestral haplotypes in the vicinity of the selected mutant and the new mutations arising on the ancestral haplotypes during the selective process. Our method adopts importance sampling algorithms to efficiently explore the genealogical history of the sample for evaluation of the sampling probability. By applying the method to both simulated and real data, we demonstrate that the extent of the haplotype structure is informative for the inference of selection intensity of a recent positive selection.

Our method has two merits. First, by exploiting the extent of haplotype structure and focusing only on subprocesses of the ARG related to the retained ancestral segments on the selected haplotypes, we dramatically reduce the computational burden such that data sets from genomic regions of mega-base magnitude can be analyzed. Second, our method can allow for changes in population size. This is especially important for samples from human populations outside Africa, because population growth can affect the pattern of linkage disequilibrium and haplotype structure, and thus lead to an incorrect estimation of the selection intensity if the effect of demographic history is not explicitly modeled. In our analysis of simulated data, we found that the estimated selection coefficient is accurate but is sensitive to the recombination rates assumed. Since the variability of recombination rates is high over human genome (Myers *et al.* 2005), good estimates of local rates are necessary to obtain accurate estimates of selection coefficients.

Another factor that may affect the estimates of selection intensity and allele age is the SNP marker density in the data. Because mutants that have experienced positive selections are typically young, new mutations at nearby loci accumulate at a relatively slow rate compared to the rate of recombination that breaks down linkage disequilibrium. Therefore, we expect fewer segregating sites observed in regions under recent positive selection. In the low-density SNP data, these segregating sites are likely not typed. In the two data sets of the *G6PD* region we analyzed, Sabeti *et al.* (2002)’s and Verrelli *et al.* (2002)’s data, no new mutations at closely linked loci were detected. Because their *G6PD* data were not generated by resequencing, a proportion of mutations may not have been identified or included in the data. We expect that resequencing data from the target gene regions will be more informative for identifying the occurrence of recombinations and mutations during the selective process. The method developed in this paper is for identifying ongoing positive selection. If the selected allele has been fixed in the population, mutations accumulated since its fixation become informative and important for inferring the fixation time, for which the allele frequency spectrum after a selective sweep is a better choice (Chen 2012).

The importance sampling algorithm for the genealogies adopted in this paper was developed by Griffiths and Tavaré (1994b). In their proposal distribution, at each step any possible event that could lead to the current configuration is considered and sampled in proportion to their rate of occurrence (Felsenstein *et al.* 1999). More efficient proposal distributions have been developed (Stephens *et al.* 2001; Slatkin 2002; De Lorio and Griffiths 2004; Paul *et al.* 2011) and can be adopted to improve the computational efficiency of our method.

## Appendix A

### The probability distribution for the extent of an intact ancestral haplotype under selection

We show how to derive the distribution for the extent of an intact ancestral haplotype at time *t* during a selective process. We investigate a selective sweep that starts from a single copy of the selected allele. Here we denote the time when the new selected mutant arises by 0, and look forward in time. Let *X_t_* be frequency of the selected allele at time *t*. If we ignore the initial randomness of allele frequency trajectories, which is usually modeled using a supercritical branching process, *X_t_* can be well approximated using the deterministic logistic equation (Ohta and Kimura, 1975):

$$X_{t}=\frac{X_{0}}{X_{0}+\left(1-X_{0}\right)e^{-st}},$$ (19)

where *X*_0_ can be set to 1/2*N* (Kaplan *et al.* 1989; Stephan *et al.* 1992). Assume the selected locus has two alleles A and a, with A being the advantageous allele. We are modeling a continuous segment between the selected mutant and the neutral marker, which has two alleles B and b. We use upper case letters to denote that the position is descended from the ancestral haplotype, and lower case letters to denote the position descended from the background haplotypes. Note here the two “alleles” are defined according to whether they are descended from the ancestral haplotype or not, instead of the true observed nucleotide types at that locus. We use [*AB*] to denote a segment of ancestral haplotype with loci A and B being the end points. Let *P*_[_*_AB_*_]_(*t*) be the relative population frequency of such fragments among all haplotypes carrying the selected allele A at time *t*. Furthermore, we use [*A*−] to denote an ancestral haplotype with *A* being from the ancestral haplotype, while the state of the other end point of the fragment is not determined.

For a random ancestral haplotype [*AB*] or [*Ab*], if it recombines with any [*A*−] haplotype during the interval [0, *t*], it does not change *P*_[_*_AB_*_]_(*t*). The only possible change is caused by “effective” recombinations, that is, recombination with an [*ab*], or [*aB*] haplotype from the neutral haplotype “sub-population”. The expected number of effective recombination events for a [*AB*] recombining with any [*a*−] haplotype during the interval [0, *t*] is

$$\begin{array}{c} C=r\int_{u=0}^{t}\left(1-X\left(u\right)\right)du\\ =rt-\frac{r}{s}\text{ln}\left(1-X_{0}+e^{st}X_{0}\right) \end{array}$$ (20)

It is not hard to see that the number of effective events on an [*AB*] ancestral haplotype during the time interval [0, *t*] follows a Poisson distribution. *P*_[_*_AB_*_]_(*t*) is then identical to the probability of no effective recombination between [*AB*] for an ancestral haplotype:

$$\begin{array}{c} P_{\left[AB\right]}\left(t\right)=e^{-rt+\frac{r}{s}\text{ln}\left(1-X_{0}+e^{st}X_{0}\right)}\\ =e^{-rt}{\left(1-\left(1-e^{st}\right)X_{0}\right)}^{r/s}. \end{array}$$ (21)

Similarly, for an ancestral haplotype [*ABC*], the probability of being intact during the interval (0, *t*) follows the Equation 21, except that the recombination fraction is replaced by *r*_[_*_AC_*_]_.

Note that in Equation 21, when *st* is small, which means either the selective process is at an early stage or the selection is weak, the term (1 − (1 − *e^st^*) *X*_0_)*^r^*^/^*^s^* ≈ 1, and thus similar to the neutral case. However, if *st* gets larger, the term cannot be ignored. For example, if *r* = 0.001, *s* = 0.01, *X*_0_ = 0.001 and *t* = 1000, the relative bias can be as large as ~27%.

Also note that in the aforementioned derivation for the distribution of ancestral haplotypes, we ignore the randomness of the frequency trajectory of the selected allele at the very early stage of the selective process, and approximate the trajectory with a deterministic equation. Ignoring the randomness of the allele frequency trajectory at the early stage can bias the inference of parameters related to the sweep process, but as pointed out in previous studies (Kaplan *et al.* 1989; Braverman *et al.* 1995; Barton, 1998; Durrett and Schweinsberg, 2004; Etheridge *et al.* 2006), when the selection intensity is sufficiently strong, the bias is small. For this reason, our method is more suitable to analyze genes under strong selections.
